# In Response to “Towards Reference Values for NT-proBNP Applicable in Pediatric Clinical Practice”

**DOI:** 10.1007/s00246-022-02937-6

**Published:** 2022-06-11

**Authors:** Alexandra Kiess, Mandy Vogel

**Affiliations:** 1 Heart Center Leipzig, Department of Pediatric Cardiology, Faculty of Medicine, University of Leipzig, Strümpellstraße 39, 04289 Leipzig, Germany; 2 LIFE Leipzig Research Center for Civilization Diseases, University of Leipzig, Philipp-Rosenthal-Strasse 27, 04103 Leipzig, Germany

**Keywords:** NT-proBNP, Age adjusted, Reference values

Dear editor and colleagues,

We appreciate the insightful comments on our article “Age-Dependent Reference Values for hs-Troponin T and NT-proBNP and Determining Factors in a Cohort of Healthy Children (The LIFE Child Study)” [1], as well as the opportunity to respond.

While we agree with Drs. Rodríguez-González and Castellano-Martínez that the Zlog values for NT-proBNP and the formula provided by Palm et al. [2] are interesting, we did not discuss their study separately because we already reviewed the underlying pooled data from four studies [3] that was used to generate the Zlog values.

We want to clarify that our percentiles are continuously estimated by age, *not* using age intervals. The data tables show point estimates for selected ages to illustrate the age dependency.

It is important to have reliable neonatal reference values; unfortunately, our study design excluded children below 3 months of age. The same applies to full echocardiographic studies, which were performed in some but not all study participants (hence, data not used).

While we have been unable to obtain the external dataset for validation, we transformed our NT-proBNP values utilizing the formula by Palm et al. [2]. The resulting Zlog scores showed a strong linear dependency compared to our standard deviation scores (SDS) [1] (*R*^2^ = 0.86, i.e., 86% explained variance from linear model) but also some deviation from each other with a strong age dependency of the Zlog values and distinct differences between SDS and Zlog: children in the first year of life had SDS greater than Zlog values; for girls, Zlog were considerably greater than SDS from the age of 3; for boys, Zlog were considerably greater than SDS between 4 and 12 years and lower from the age of 15 (Fig. 1). As expected, there was no age dependence of the SDS values, because the references originated from the same data. A real test was not feasible because of lacking external data. The differences between the Zlog scores and our SDS could be explained by the different cohort compositions: patient vs. healthy cohort, non-consideration of sex (especially since we found significant sex differences), and assumption of a functional relationship that might be too strong.

**Fig. 1:**
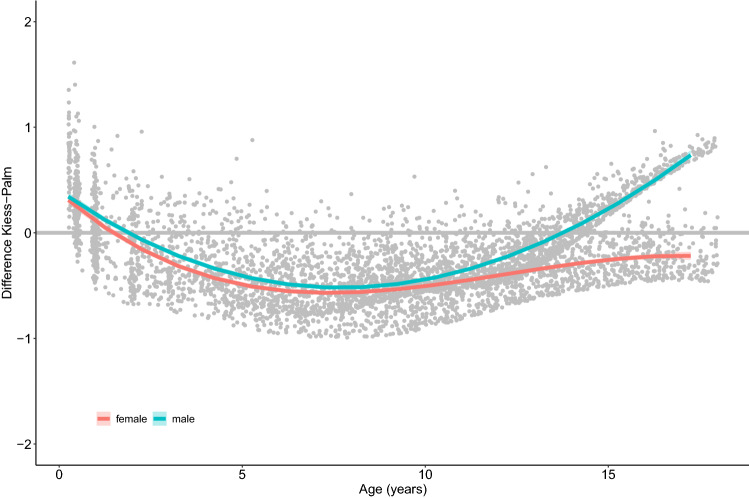
Differences between SDS (standard deviation scores) and Zlog utilizing the formula by Palm et al. [2] of NT-proBNP values of the LIFE Child dataset [1]. Children in the first year of life had greater SDS than Zlog. Girls (red) had considerably greater Zlog than SDS from 3 years of age, while in boys (blue) Zlog were considerably greater than SDS values between 4 and 12 years, but lower from the age of 15. Differences could be explained by the different cohort compositions, differentiation in males and females, and too high assumption of a functional relationship

In conclusion, we found the model by Palm et al. similar but not entirely fitting to our dataset^1^ since there was still a strong age dependency after transformation, as well as sex differences. Further validation of both studies as well as discussion regarding whether Zlog values or SDS would be more clinically useful is warranted.

## Supplementary Information

Below is the link to the electronic supplementary material.

Supplementary file1 (PDF 75 kb)

## Footnotes

1 We put the references to an R package (https:/cran.r-project.org/package = childsds) to make them easily usable.

## References

[1] Kiess A, Green J, Willenberg A, Ceglarek U, Dähnert I, Jurkutat A, Körner A, Hiemisch A, Kiess W, Vogel M (2022). Age-dependent reference values for hs-troponin T and NT-proBNP and determining factors in a cohort of healthy children (The LIFE Child Study). Pediatr Cardiol.

[2] Palm J, Hoffmann G, Klawonn F, Tutarel O, Palm H, Holdenrieder S, Ewert P (2020). Continuous, complete and comparable NT-proBNP reference ranges in healthy children. Clin Chem Lab Med.

[3] Nir A, Lindinger A, Rauh M, Bar-Oz B, Laer S, Schwachtgen L, Koch A, Falkenberg J, Mir TS (2009). NT-pro-B-type natriuretic peptide in infants and children: reference values based on combined data from four studies. Pediatr Cardiol.

