# Supplementary material for: In Response to “Towards Reference Values for NT-proBNP Applicable in Pediatric Clinical Practice”
Source: Pediatr Cardiol. 2022 Jun 11;43(6):1405–6. doi: 10.1007/s00246-022-02937-6 (PMC9294008; doi:10.1007/s00246-022-02937-6)
Supplement: Supplementary file 1 — Supplementary file1 (PDF 75 kb) [file 246_2022_2937_MOESM1_ESM.pdf]

Table 2: Estimated 5th, 50th, 75th, 95th and 97.5th sex-adjusted percentiles for NT-proBNP (values in ng/L) from the LIFE child study cohort (children aged 0.25-18 years) in comparison to the non sex-specific 75th and 97.5th percentiles from Albers et al. [4]

LIFE data NT-proBNP (ng/L) select percentiles													75th and 97.5th percentiles acc. Albers et al. [4]			
age, years	n		5th		50th		75th		95th		97.5th		age, years	n	75th	97.5th
	female	male	female	male	female	male	female	male	female	male	female	male				
0,25	77	77	54,3	46,7	157,8	139,7	230,8	204,1	388,5	345,2	**458,8**	**409,9**				
0,5	92	98	50,6	44,1	147,2	133,0	215,6	194,7	363,5	330,3	**429,4**	**392,4**				
1	92	121	44,1	39,8	129,4	121,5	190,0	178,6	321,2	304,6	**379,7**	**362,6**	1—3	13	231.2	**319.9**
2	81	98	35,5	33,5	105,5	105,3	155,5	156,2	264,4	269,2	**313,1**	**321,5**				
3	75	82	30,8	29,2	93,0	94,6	137,7	141,5	235,4	246,7	**279,3**	**295,6**				
4	79	77	28,6	26,0	87,5	87,0	130,2	131,3	223,8	231,4	**265,9**	**278,3**	4—6	21	112.6	**189.7**
5	98	104	27,4	24,0	85,2	82,9	127,3	126,3	220,1	225,3	**262,0**	**271,8**				
6	113	115	26,5	22,9	83,5	81,6	125,4	125,4	218,1	226,2	**260,1**	**273,9**				
7	128	130	25,4	21,2	81,3	78,3	122,6	121,5	214,5	221,9	**256,2**	**269,6**	7—9	32	94.4	**144.7**
8	136	162	23,9	18,7	77,8	71,6	117,8	112,2	207,4	207,3	**248,1**	**252,8**				
9	146	178	22,2	16,1	73,1	63,8	111,3	101,0	197,0	189,0	**236,2**	**231,3**				
10	155	174	19,9	14,1	66,8	57,9	102,1	92,7	181,9	175,6	**218,4**	**215,7**	10	11	72.5	**112.4**
11	154	161	17,6	12,8	59,8	54,8	91,9	88,5	164,7	169,8	**198,1**	**209,4**	11	69	93.4	**317.1**
12	158	174	15,4	11,5	53,3	50,9	82,3	83,1	148,4	161,6	**178,9**	**200,1**	12	21	95.0	**186.4**
13	163	160	13,6	9,5	48,0	43,8	74,6	72,3	135,2	142,4	**163,3**	**177,0**	13	23	113.6	**369.9**
14	162	168	12,3	7,2	44,1	34,8	68,9	58,1	125,7	115,9	**152,0**	**144,6**	14	18	68.2	**362.8**
15	130	117	11,3	5,3	41,2	26,7	64,6	45,1	118,6	91,3	**143,7**	**114,4**	15	24	73.6	**216.7**
16	127	100	10,5	4,0	39,0	20,8	61,4	35,6	113,5	73,0	**137,8**	**91,8**	16	24	84.9	**206.0**
17	78	50	10,1	3,1	37,9	16,8	60,1	29,0	111,7	60,3	**135,9**	**76,2**	17	24	71	**134.9**

Table 3: Estimated 5th, 50th, 75th, 95th and 97.5th sex-adjusted percentiles for hs-Troponin T (values in ng/L) from the LIFE child study cohort (children aged 0.25-18 years)

LIFE data hs-Troponin T (ng/L) select percentiles

age, years	n		5th		50th		75th		95th		97.5th	
	female	male	female	male	female	male	female	male	female	male	female	male
0.25	20	17	8,35	6,11	17,71	16,22	22,25	20,47	32,38	28,26	37,72	31,66
0.5	79	89	5,14	3,92	10,81	10,12	13,51	12,71	19,67	17,49	23,04	19,61
1	88	107	2,56	2,05	5,32	5,04	6,58	6,26	9,63	8,58	11,43	9,64
2	77	83	1,73	1,50	3,59	3,45	4,37	4,21	6,52	5,75	8,02	6,50
3	74	76	1,39	1,44	2,90	3,15	3,49	3,80	5,33	5,19	6,83	5,93
4	74	73	1,39	1,37	2,91	2,93	3,47	3,50	5,41	4,81	7,18	5,56
5	93	102	1,36	1,42	2,81	2,99	3,31	3,55	5,20	4,94	7,05	5,80
6	111	111	1,28	1,48	2,58	3,12	3,02	3,69	4,70	5,21	6,36	6,24
7	118	127	1,36	1,40	2,64	2,96	3,08	3,49	4,67	5,04	6,20	6,16
8	130	155	1,51	1,28	2,81	2,75	3,25	3,24	4,79	4,78	6,18	5,98
9	142	173	1,53	1,28	2,74	2,81	3,16	3,33	4,52	5,02	5,65	6,41
10	149	174	1,62	1,22	2,80	2,74	3,22	3,26	4,49	5,04	5,47	6,54
11	152	155	1,66	1,30	2,81	3,01	3,23	3,60	4,42	5,66	5,27	7,45
12	157	172	1,66	1,25	2,77	3,00	3,19	3,61	4,31	5,78	5,07	7,67
13	158	154	1,62	1,25	2,71	3,12	3,13	3,80	4,21	6,16	4,91	8,18
14	156	168	1,71	1,23	2,88	3,25	3,34	4,01	4,51	6,55	5,26	8,66
15	123	105	1,72	1,30	2,97	3,67	3,47	4,59	4,75	7,54	5,57	9,88
16	122	94	1,61	1,38	2,93	4,22	3,45	5,38	4,83	8,87	5,77	11,48
17	74	48	1,45	1,37	2,84	4,64	3,39	6,04	4,97	10,00	6,14	12,77
18	36	21	1,18	1,56	2,63	5,98	3,18	8,00	5,07	13,31	6,71	16,76
